# Characterization of a Lamellocyte Transcriptional Enhancer Located within the *misshapen* Gene of *Drosophila melanogaster*

**DOI:** 10.1371/journal.pone.0006429

**Published:** 2009-07-29

**Authors:** Tsuyoshi Tokusumi, Richard Paul Sorrentino, Mark Russell, Roberto Ferrarese, Shubha Govind, Robert A. Schulz

**Affiliations:** 1 Department of Biological Sciences, University of Notre Dame, Notre Dame, Indiana, United States of America; 2 Department of Biochemistry and Molecular Biology, University of Texas M. D. Anderson Cancer Center, Houston, Texas, United States of America; 3 Department of Biology, City University of New York, New York, New York, United States of America; Universidade Federal do Rio de Janeiro (UFRJ), Instituto de Biofísica da UFRJ, Brazil

## Abstract

*Drosophila* has emerged as an excellent model system in which to study cellular and genetic aspects of hematopoiesis. Under normal developmental conditions and in wild-type genetic backgrounds, *Drosophila* possesses two types of blood cells, crystal cells and plasmatocytes. Upon infestation by a parasitic wasp or in certain altered genetic backgrounds, a third hemocyte class called the lamellocyte becomes apparent. Herein we describe the characterization of a novel transcriptional regulatory module, a lamellocyte-active enhancer of the *misshapen* gene. This transcriptional control sequence appears to be inactive in all cell types of the wild-type larva, including crystal cells and plasmatocytes. However, in lamellocytes induced by wasp infestation or by particular genetic conditions, the enhancer is activated and it directs reporter GFP or DsRed expression exclusively in lamellocytes. The lamellocyte control region was delimited to a 140-bp intronic sequence that contains an essential DNA recognition element for the AP-1 transcription factor. Additionally, mutation of the *kayak* gene encoding the dFos subunit of AP-1 led to a strong suppression of lamellocyte production in tumorous larvae. As *misshapen* encodes a protein kinase within the Jun N-terminal kinase signaling pathway that functions to form an active AP-1 complex, the lamellocyte-active enhancer likely serves as a transcriptional target within a genetic auto-regulatory circuit that promotes the production of lamellocytes in immune-challenged or genetically- compromised animals.

## Introduction

Blood cell production, also termed hematopoiesis, is a highly conserved developmental process. Though separated by millions of years of evolution, striking similarities exist between hematopoiesis in *Drosophila* and humans [1], [2]. Such similarities include multiple sites and times of blood cell production, the generation of functionally distinct cell types from common hematopoietic precursors, and the utilization of conserved signaling pathways and transcriptional regulators for pro-hemocyte determination and specific lineage differentiation. Furthermore, hemocyte populations are derived from progenitor hemangioblasts that give rise to multiple cell fates within the mesoderm of both developmental systems [3]. Thus due to this evolutionary conservation and the rapid and sensitive approaches available, *Drosophila* has emerged as a leading model system to discover and analyze genes controlling hematopoiesis. Blood cell production in *Drosophila* occurs in two spatiotemporal waves. Using blastoderm stage embryos, Holz and co-workers [4] carried out single-cell transplantation experiments and demonstrated pro-hemocytes arise from two different embryonic anlagen. These are the cephalic mesoderm that gives rise to embryonic hemocytes, and a thoracic mesodermal region that will form the lymph glands, the organ for hematopoiesis during larval development [5]. *Drosophila* hemocytes possess characteristics similar to those blood cells found in vertebrate myeloid lineages [6]. Embryo-derived types include crystal cells and plasmatocytes [1], [2]. Crystal cells constitute the minor class of hemocytes and carry crystals of Prophenoxidase A1, a pro-enzyme that is processed to form an active enzyme required for catalyzing melanin synthesis during wound healing and encapsulation reactions. Prophenoloxidase A1 is encoded by *Black cells* (*Bc*), a gene expressed primarily in crystal cells [7]. In contrast, plasmatocytes constitute the major class of hemocytes and their chief function is in phagocytosis, engulfing apoptotic corpses formed as a result of embryonic tissue development. Plasmatocytes also secrete and remodel extracellular matrix important for animal development and rid the animal of certain small invading pathogens. Eater is a transmembrane protein that is expressed solely in post-embryonic plasmatocytes where it mediates phagocytosis of bacterial pathogens [8].

Embryo-derived blood cells persist into larval stages, where they undergo multiple rounds of mitosis. By the end of the third larval instar, about 6–8,000 hemocytes are observed in the circulating hemolymph [9]. Crystal cells and plasmatocytes are also produced in the lymph gland during second and third larval instars, but these hemocytes remain sequestered in the hematopoietic organs and not contributed to circulation until metamorphosis. At that time, they are released into the hemolymph, to assist in the histolysis of larval tissues. Hematopoietic organs have not been identified in adult *Drosophila*. Blood cells found in adult flies represent a mixed population of hemocytes with embryonic or larval origins.

Lamellocytes constitute a third class of hemocytes, that when induced, appear in the lymph glands and hemolymph during larval development. These blood cells are large and flat with adhesive properties, and they function to encapsulate and neutralize objects that are too large to be phagocytized by plasmatocytes. Lamellocyte induction can occur due to an immune response to wasp parasitization [10], [11]. It was recently shown that lamellocytes that encapsulate wasp eggs are derived mainly from a subepidermal population of sessile hemocytes initially found in posterior segments of the larva [12]. Lamellocytes can also be induced through genetic perturbation of specific signal transduction pathways that can result in melanotic tumor phenotypes [11]. Such genetic backgrounds include mutation or forced expression of genes functioning in the JAK/STAT, Jun N-terminal kinase (JNK), Toll, and Wingless (Wg) pathways [13]. Additionally, loss-of-function of the *u-shaped* (*ush*) gene, which encodes a Friend of GATA class protein, culminates in a robust production of lamellocytes [9].

The precise mechanisms of how these different signaling pathways or transcriptional regulators function and/or interact in lamellocyte induction and differentiation remain to be elucidated. Nonetheless, some progress has been made in investigating the role of the JAK/STAT pathway in regulating lamellocyte production. The gain-of-function allele *hop^Tum-l^* encodes a constitutively active form of the Hopscotch (Hop) Janus kinase (JAK) and larvae carrying this mutation exhibit a melanotic tumor phenotype including extensive lamellocyte differentiation [14]. Consistently, loss of function of Hop almost completely suppressed the differentiation of larval lymph gland lamellocytes and the appearance of lamellocytes in circulation in response to wasp parasitization [15]. More recent studies demonstrated that JAK over-activation results in a global disruption of heterochromatic gene silencing, with a de-repression of genes not normally serving as targets of STAT regulation leading to enhanced tumorigenesis [16]. Forced expression of the Ush transcriptional co-regulator in *hop^Tum-l^* larvae results in a 90% reduction of the population of circulating lamellocytes, indicating Ush plays a central role in the suppression of lamellocyte differentiation [9].

Two known markers for the lamellocyte lineage are an antigen recognized by the L1 monoclonal antibody [17] and the enhancer trap allele *misshapen^03349^* (*msn^03349^*) [18]. Msn is a MAPK kinase kinase kinase (MAPKKKK) within the JNK signaling cascade, a pathway known to function in lamellocyte production [13] and in cell migration, cytoskeleton rearrangement and cell shape change, and tissue morphogenesis [19]. Based on the ability of sequences near or within the *msn* locus to direct *PlacZ* transgene expression in lamellocytes, we initiated an analysis of this gene in search of its lamellocyte-active transcriptional enhancer. In this report, we characterize an intronic regulatory module that functions solely in lamellocytes, being regulated by the AP-1 transcription factor. These studies provide further molecular and genetic evidence on the importance of the *msn* gene and JNK pathway signaling in lamellocyte formation. They have also yielded highly-sensitive *msn-GFP* and *msn-DsRed* transgenes that can be used to monitor the induction and function of lamellocytes in immune-challenged or genetically-altered *Drosophila*.

## Results

### A lamellocyte-active enhancer present within intron-3 of the *msn* gene

**Figure 1:**
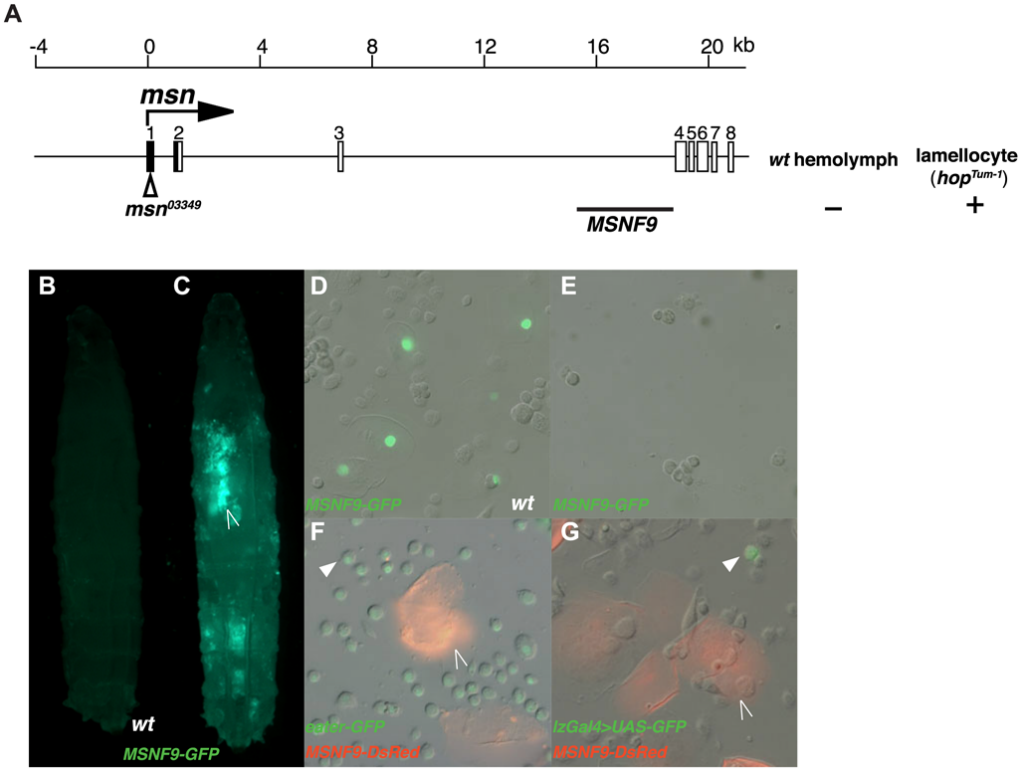
Identification and initial characterization of a *msn* lamellocyte transcriptional enhancer. *A*. Genomic organization of the *msn* gene and location of the 3.0-kb MSNF9 intron-3 DNA that possesses lamellocyte enhancer activity. *B*. Lack of *MSNF9-GFP* expression in a wild-type third-instar larva. *C*. The *MSNF9-GFP* transgene is active in a *hop^Tum-l^* third-instar larva. The open arrowhead points to *MSNF9-GFP*-positive lamellocytes that have contributed to the formation of a melanotic tumor in this animal. *D*. *MSNF9-GFP* activity is not detected in circulating hemocytes present in hemolymph obtained from a wild-type third-instar larva. *E*. *MSNF9-GFP*-positive lamellocytes present in hemolymph obtained from a *hop^Tum-l^* third-instar larva. *F*. Lack of co-expression of *eater-GFP* (plasmatocyte marker, arrowhead) and *MSNF9-DsRed* (lamellocyte marker, open arrowhead) in circulating blood cells obtained from a *hop^Tum-l^* third-instar larva. *G*. Lack of co-expression of *lzGal4>UAS-GFP* (crystal cell marker, arrowhead) and MSNF9-DsRed (lamellocyte marker, open arrowhead) in circulating blood cells obtained from a *hop^Tum-l^* third-instar larva.

*msn^03349^* is a *PlacZ* enhancer trap allele of *msn* that results in β-galactosidase expression in lamellocytes induced in larvae due to wasp parasitization [11], [15], [18]. Based on this finding, we initiated a series of experiments to localize and characterize a transcriptional control sequence contributing to this selective hemocyte expression. Enhancer analyses were initiated wherein ∼22 kb of *msn* upstream or intronic DNAs were cloned into P-element vectors containing GFP or DsRed reporter genes and transgenic strains were established that harbor the nine DNA-marker transgenes. Wild-type larvae possessing the transgene insertions were monitored for GFP or DsRed expression in blood cells present within the lymph glands or hemolymph. To determine if *msn* DNAs possessed enhancer activity in lamellocytes, expression of the various transgenes was assayed after being crossed into the *hop^Tum-l^* genetic background. MSNF9 (Fig. 1A) is a 3.0-kb region of *msn* intron-3 possessing the desired characteristic of being active in lamellocytes induced in *hop^Tum-l^* animals (Fig. 1C and E), while being inactive in other hematopoietic cell types within wild-type larva (Fig. 1B and D). Comparing the cellular activity of *MSNF9-DsRed* with the *eater-GFP* plasmatocyte (Fig. 1F) or *lzGal4>UAS-GFP* crystal cell (Fig. 1G) markers demonstrated the transcriptional enhancer within MSNF9 functioned solely in lamellocytes and not in the other two blood cell types.

**Figure 2:**
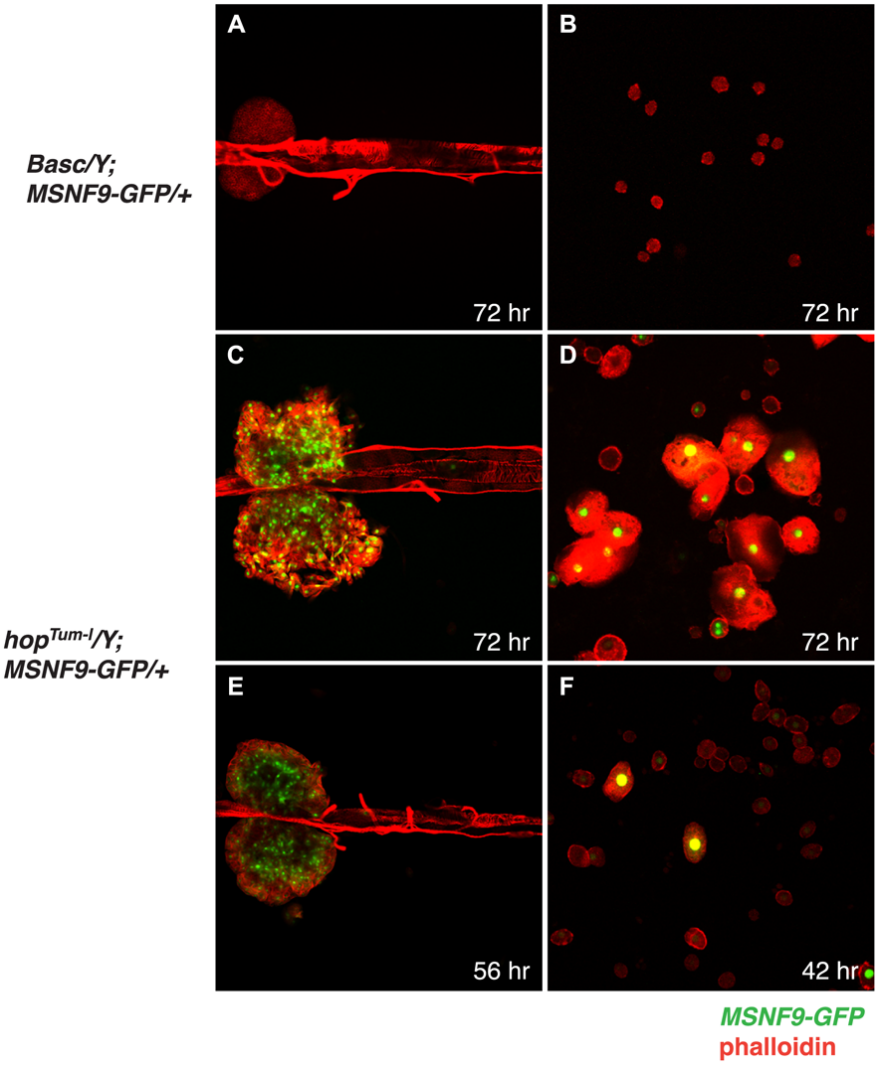
Temporal activation of the *msn* lamellocyte transcriptional enhancer. *A*. Inactivity of the *MSNF9-GFP* transgene in lymph glands dissected from a *Basc/Y* control larva at 72 hr of development. *B*. Inactivity of the *MSNF9-GFP* transgene in circulating hemocytes present in hemolymph obtained from a *Basc/Y* control larva at 72 hr of development. *C*. *MSNF9-GFP*-positive hemocytes observed in lymph glands dissected from a *hop^Tum-l^/Y* mutant larva at 72 hr of development. *D*. *MSNF9-GFP*-positive lamellocytes present in the hemolymph obtained from a *hop^Tum-l^/Y* mutant larva at 72 hr of development. *E*. *MSNF9-GFP*-positive hemocytes observed in lymph glands dissected from a *hop^Tum-l^/Y* mutant larva at 56 hr of development. *F*. *MSNF9-GFP*-positive lamellocytes present in the hemolymph obtained from a *hop^Tum-l^/Y* mutant larva at 42 hr of development.

To determine when the MSNF9 lamellocyte enhancer initiated transcriptional function, we conducted tightly-timed egg lays and aging of control and *hop^Tum-l^* animals that harbored the *MSNF9-GFP* transgene. Lymph glands dissected from *Basc/Y;MSNF9-GFP/+* control larvae at 72 hr of development failed to exhibit any lamellocyte differentiation or GFP expression (Fig. 2A). In contrast, lymph glands obtained from *hop^Tum-l^/Y; MSNF9-GFP/+* test larvae at 72 hr of development showed lymph gland hyperplasia, extensive lamellocyte differentiation, and robust GFP expression (Fig. 2C). The analysis of multiple larvae indicated that *hop^Tum-l^*-induced lamellocytes reproducibly appeared in the lymph glands around 54–56 hr of development at 25°C (Fig. 2E). As expected, the analysis of hemolymph samples taken from control larvae aged to 72 hr showed an absence of lamellocytes and GFP-positive cells (Fig. 2B). In contrast, a large population of GFP-positive lamellocytes was observed in hemolymph obtained from *hop^Tum-l^* larvae at 72 hr of development (Fig. 2D). Moving back from this time point, *MSNF9-GFP*-positive hemocytes reproducibly appeared in these mutant larvae before 56 hr of development at 25°C, with rare positive hemocytes being detected in the hemolymph of *hop^Tum-l^* animals as early as 42 hr of development (Fig. 2F). It is likely these early-appearing *MSNF9-GFP*-positive hemocytes serve as precursors of the later appearing lamellocyte population. Thus the *msn* transcriptional enhancer is functional in blood cells within the hemolymph before becoming active in cells of the lymph glands.

**Figure 3:**
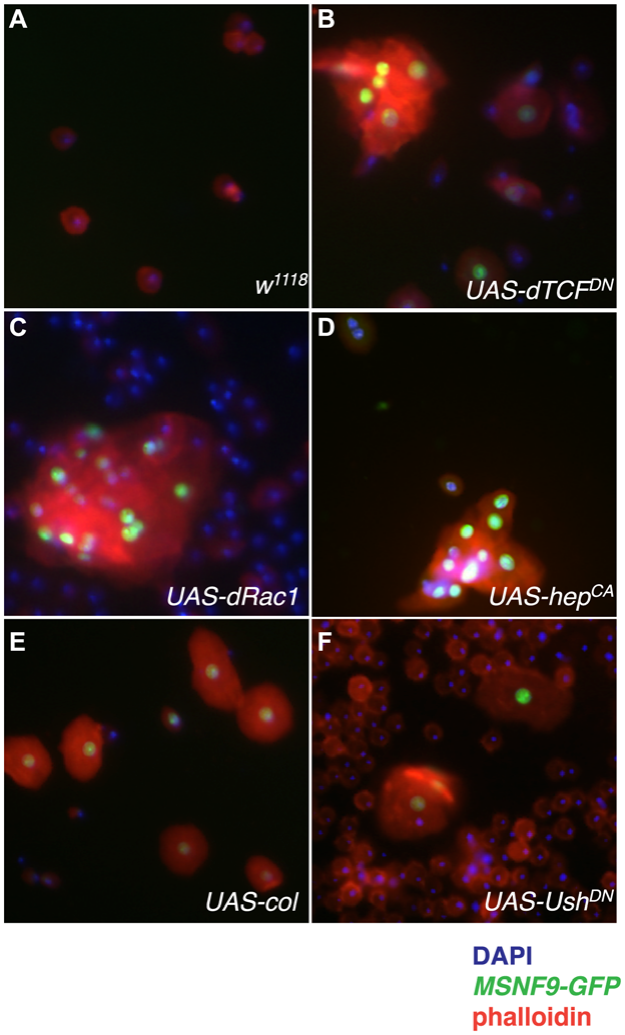
*MSNF9-GFP* expression in circulating hemocytes obtained from third-instar larvae of different genetic backgrounds that induce lamellocyte production. *A*. *w^1118^; He-Gal4* control. *B*. *He-Gal4>UAS-dTCF^DN^*. *C*. *He-Gal4>UAS-dRAC1*. *D*. *He-Gal4>UAS-hep^CA^*. *E*. *Cg-Gal4>UAS-col*. *F*. *Cg-Gal4>Ush^DN^*. Hemolymph samples were also stained for DNA (DAPI) and filamentous actin (phalloidin).

Lamellocyte production can be induced in a variety of genetic backgrounds, including in larvae with forced hemocyte expression of a dominant-negative version of dTCF (dTCF^DN^) [13], a transcriptional effector of the Wg pathway, and forced expression of dRAC1 or a constitutively-active version of Hemipterous (hep^CA^) [13], two members of the JNK signaling pathway. Likewise, forced hemocyte expression of transcriptional regulators such as Collier (Col) [20] or a dominant-negative version of Ush (Ush^DN^) [9] culminate in precocious lamellocyte production. An analysis of *MSNF9-GFP* expression in larvae of these five different genotypes demonstrated the *msn* enhancer is active in lamellocytes induced due to all of these genetic conditions (Fig. 3B–F). These findings demonstrated the usefulness of the *MSNF9-GFP* transgene for the detection of cells entering this specific hemocyte lineage.

### The *msn* lamellocyte enhancer functions in larvae mounting an immune response to wasp parasitization

**Figure 4:**
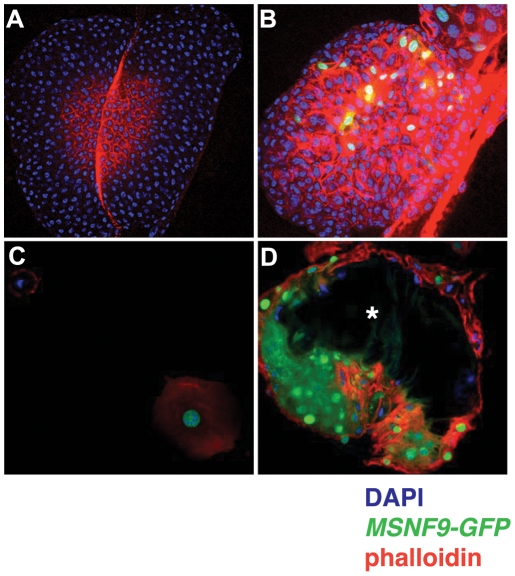
*MSNF9-GFP* expression in larvae post *L. boulardi* infestation. *A*. Lack of *MSNF9-GFP* activity in lymph glands dissected from a control, non-wasp infested larva. *B*. *MSNF9-GFP* expression in lymph glands dissected from a wasp infested larva. *C*. *MSNF9-GFP*-positive lamellocyte present in the hemolymph of a wasp infested larva. *D*. Detection of numerous *MSNF9-GFP*-positive lamellocytes encapsulating a wasp egg (asterisk).

In response to parasitoid wasp infestation, larvae rapidly generate lamellocytes that function to encapsulate and neutralize a deposited egg [10], [11]. To determine if the MSNF9 enhancer was active in lamellocytes induced under these more natural conditions, wasps were incubated with larvae carrying the *MSNF9-GFP* transgene. We initially monitored the induction of GFP activity in lymph glands dissected from larvae incubated without or with the parasite. While the *MSNF9-GFP* transgene was not activated in the hematopoietic organs of control larvae (Fig. 4A), the lamellocyte marker was induced in lymph glands obtained from wasp-infested animals (Fig. 4B). Wasp parasitization likewise resulted in the production of *MSNF9-GFP*-positive lamellocytes within the hemolymph (Fig. 4C) and the generation of copious numbers of lamellocytes that encapsulated a deposited egg (Fig. 4D). These findings demonstrated the *msn* transcriptional control sequence was functional in lamellocytes induced by a natural immune-challenge as well as specific alterations of genetic background.

### Detailed molecular analysis of the *msn* lamellocyte regulatory module

**Figure 5:**
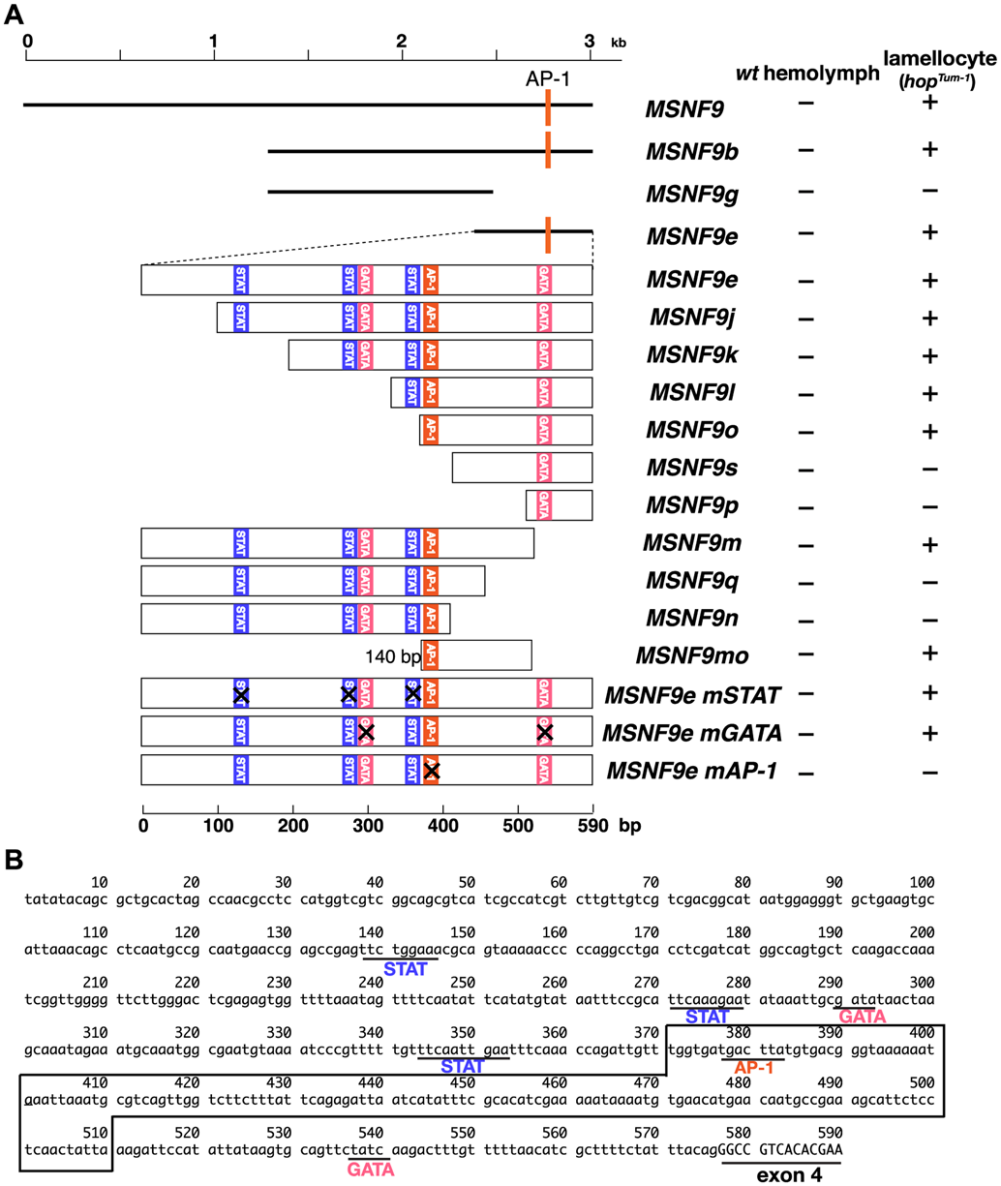
Fine mapping of the location of the *msn* lamellocyte enhancer and its essential DNA sequence elements. *A*. Schematic of the various truncated and sequence mutated *msn* intron-3 DNAs tested for enhancer function in lamellocytes of *hop^Tum-l^* larvae as compared to hemocytes of control larvae. *B*. Sequence of the 590-bp MSNF9e DNA with putative binding elements for the STAT, Srp, and AP-1 transcription factors highlighted.

Having demonstrated the 3.0-kb MSNF9 interval of *msn* intron-3 harbored a lamellocyte-active enhancer, we sought to more precisely locate the transcriptional control sequence while also investigating its means of regulation. As a start, three truncated versions of MSNF9 were generated: MSNF9b, MSNF9e, and MSNF9g (Fig. 5A). The first two represent 5′-truncations of MSNF9 and they both maintained enhancer activity in lamellocytes. In contrast, MSNF9g deletes both 5′- and 3′-sequences from MSNF9, alterations that led to a loss of lamellocyte enhancer function. Taken together, these initial delimitation analyses allowed us to refine the location of the *msn* lamellocyte enhancer to the 590-bp MSNF9e DNA.

The next set of lamellocyte enhancer test constructs involved the generation and analysis of ten truncated versions of MSNF9e, with the positions of the PCR primers carefully chosen based on the locations of potential binding sites for the STAT, Srp, or AP-1 transcription factors (Fig. 5A). The MSNF9mo DNA was the smallest region that maintained lamellocyte enhancer function in these analyses. Thus we were able to delimit the regulatory module to a 140-bp region of *msn* intron-3, a sequence that lacked putative STAT and Srp binding elements but possessed an evolutionarily conserved AP-1 binding element. To determine if this putative AP-1 recognition element was essential for lamellocyte activity of the enhancer, we mutated this sequence within the context of the 590-bp MSNF9e DNA to generate the altered MSNF9e-mAP-1 construct. The precise mutation of this putative AP-1 binding site fully abrogated lamellocyte enhancer function, strongly implicating the AP-1 transcription factor complex as a positive regulator of this minimally-defined *msn* lamellocyte regulatory module.

**Figure 6:**
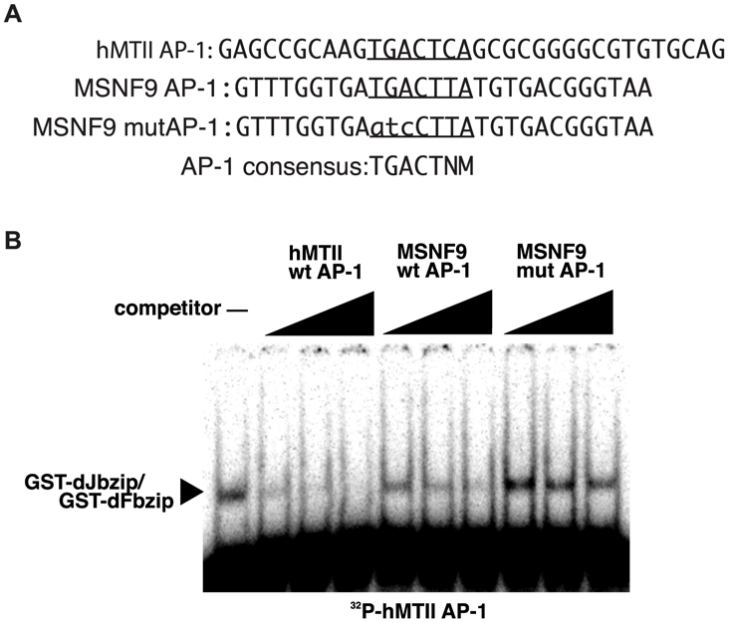
The AP-1 recognition sequence in the *msn* lamellocyte enhancer can compete for dFos-dJun DNA binding. *A*. Sequence of the hMTII AP-1 DNA probe, MSNF9 AP-1 recognition site, and mutated MSNF9 AP-1 recognition site. *B*. Electromobility shift competition assay with hMTII wt AP-1, MSNF9 wt AP-1, and MSNF9 mut AP-1 double-stranded oligonucleotide DNAs.

Based on this mutagenesis and enhancer test result, electromobility shift assays were undertaken to test the ability of the combination of dFos (encoded by *kayak*, *kay*) and dJun (encoded by *Jun-related antigen*, *Jra*) DNA binding domain proteins to interact with a double-stranded oligonucleotide containing the AP-1 recognition element. We were unable to demonstrate a strong interaction of the truncated proteins with the required sequence present in the *msn* lamellocyte enhancer (data not shown), suggesting an additional protein(s) may be needed to facilitate or stabilize this protein-DNA interaction. As a second means to test the ability of this dFos/dJun combination to interact with the essential enhancer sequence, we were able to show the *Drosophila* proteins could complex with a high-affinity AP-1 binding site present in the hMTII promoter [21] (Fig. 6A and B). Formation of this protein-DNA complex was inhibited by increasing amounts of a wild-type DNA containing the *msn* enhancer AP-1 recognition site, but not with a mutated version of this site (Fig. 6A and B). We concluded the *msn* lamellocyte enhancer AP-1 sequence could compete for dFos/dJun binding in this electromobility shift competition assay, again implicating a role for the AP-1 complex in the positive regulation of enhancer activity.

### Mutation of the gene encoding *Drosophila* Fos strongly suppresses lamellocyte production

**Figure 7:**
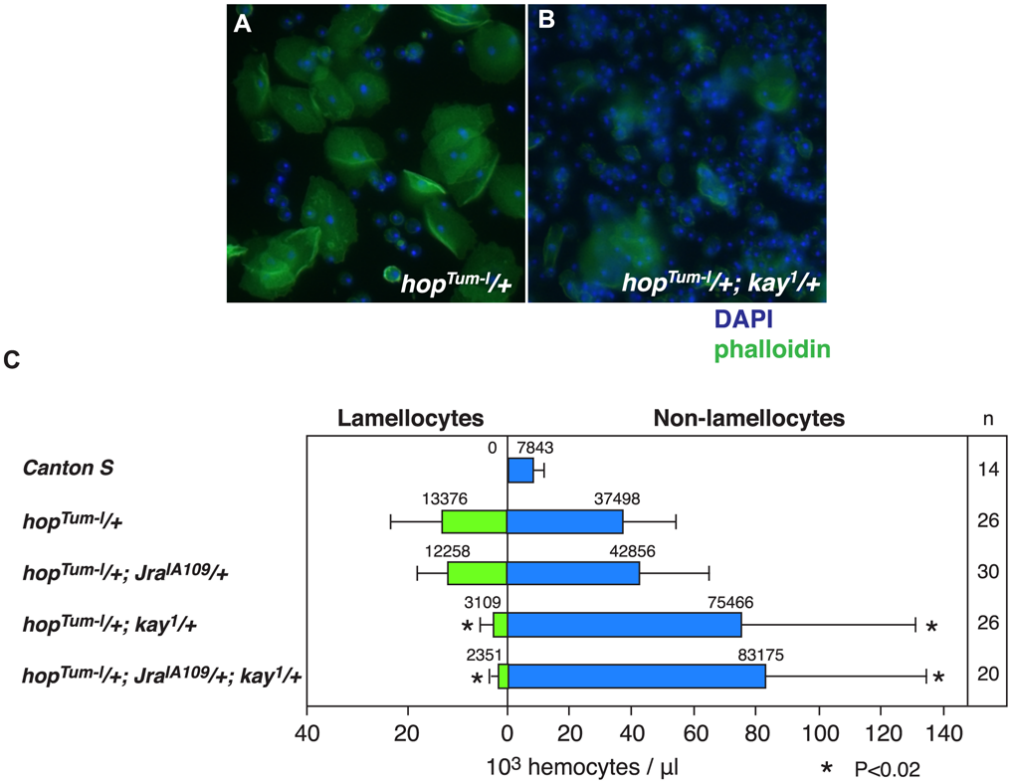
Suppression of lamellocyte production by a mutant allele of *kay*, which encodes dFos. *A*. Supernumerary lamellocytes produced in a *hop^Tum-l^/+* larva. *B*. Substantial reduction in the lamellocyte population in a *hop^Tum-l^/+*; *kay^1^/+* larva. *C*. Quantification of the number of lamellocytes and non-lamellocytes produced in larva of *Canton S* control versus mutant larvae. Asterisk (*) denotes a significant difference in values from those observed in *hop^Tum-l^* animals.

As noted previously, the *hop^Tum-l^* mutation results in a massive overproduction of both lamellocytes and non-lamellocyte hemocytes in larvae as compared to similarly-staged wild-type animals (Fig. 7C). To investigate if mutations in the genes encoding dJun or dFos proteins could lead to altered levels of lamellocyte differentiation and/or hemocyte proliferation, we monitored blood cell production in *hop^Tum-l^* larvae that were also heterozygous mutant for amorphic alleles of *Jra* and/or *kay*. While the introduction of the *Jra* mutant allele showed a small affect on the level of lamellocyte production and hemocyte proliferation as induced by *hop^Tum-l^*, introducing the *kay* mutant allele resulted in a robust 77% suppression in the number of lamellocytes produced (Fig. 7A–C). Introducing a *Jra* mutant allele to generate a *hop^Tum-l^/+*; *Jra^IA109^/+*; *kay^1^/+* triple mutant condition led to an apparent further reduction in lamellocyte production. However, statistical t-test analyses showed no significant difference in the number of lamellocytes in double and triple mutant larvae. We concluded that reducing dFos protein activity has a dramatic effect on lamellocyte production in *hop^Tum-l^* animals, also that decreasing dFos function enhances the hyper-proliferative effect of the *hop^Tum-l^* mutation. Such a finding is consistent with a function of the AP-1 complex in the regulation of the *msn* lamellocyte enhancer during the specification and differentiation of this induced blood cell type.

## Discussion

The *msn* ^03349^ enhancer trap allele has served as a valuable marker for lamellocyte induction and differentiation [11], [15], [18]. This finding led us to a thorough search of *msn* upstream and intragenic DNA for a transcriptional regulatory module with activity in this inducible class of hemocytes. MSNF9 was shown to contain DNA sequences that directed reporter gene expression exclusively in lamellocytes, but not crystal cells or plasmatocytes of *hop^Tum-l^* larvae. Furthermore, this transcriptional enhancer was functional in lamellocytes induced in several altered genetic backgrounds, such as forced expression of the JNK pathway components *dRAC1* and *hep^CA^*, the Wg pathway component *dTCF^DN^*, and the transcriptional regulators *col* and *ush^DN^*. Importantly, *MSNF9-GFP*-positive cells were induced in the lymph glands and hemolymph of larvae infested by *L. boulardi* parasitoid wasps, with numerous fluorescent lamellocytes contributing to the encapsulation of injected eggs. Together these results demonstrated the identified *msn* enhancer had the properties of being selectively activated in both genetically-compromised and immune-challenged animals, wherein it served as a discriminating sensor of lamellocyte production.

An interesting characteristic of the *msn* regulatory module is it becomes functional in lamellocytes produced in the hemolymph several hours prior to lamellocytes being detected in the lymph glands of *hop^Tum-l^* larvae. This earlier hemolymph appearance of *MSNF9-GFP*-positive cells is consistent with lamellocytes being derived from an extant population of hemocytes within the hemolymph, such as the plasmatocyte sub-group of blood cells [9], [22]. This differential spatial and temporal appearance of lamellocytes makes further sense in light of a recent study that concluded during the onset of an immune response to wasp parasitization, lamellocytes were derived from sessile hemocytes initially found in a posterior hematopoietic compartment of the larva [12]. These investigations also demonstrated lymph gland-derived lamellocytes appeared at a later stage of the immune response, a time when they were likely to augment the encapsulation of the introduced wasp egg.

**Figure 8:**
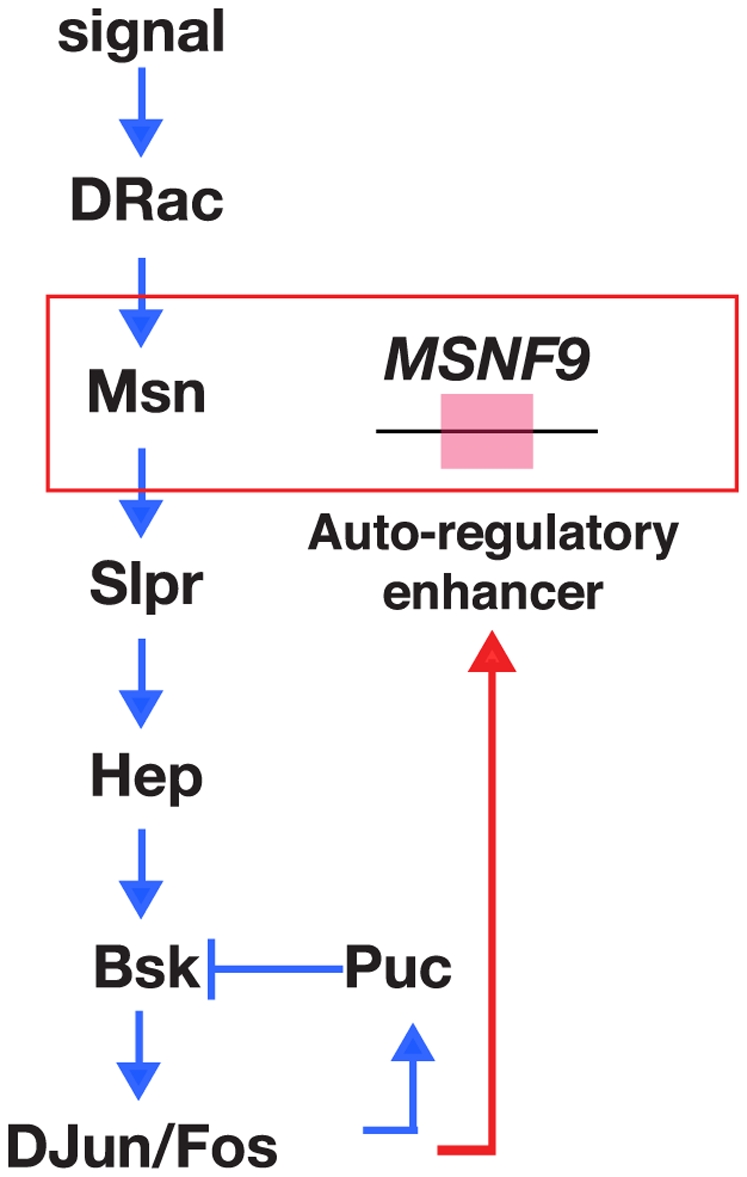
Model for the regulation of the *msn* gene during lamellocyte induction and differentiation. The MSNF9 lamellocyte-active enhancer is depicted as serving as a transcriptional target within a JNK pathway auto-regulatory circuit that promotes the production of lamellocytes in immune-challenged or genetically compromised animals. The schematic of the JNK pathway is adapted from Xia and Karin (19).

Our in depth molecular analysis of the lamellocyte enhancer allowed us to delimit the regulatory module to a 140-bp interval of *msn* intron-3. This minimal DNA region was shown to possess an essential DNA recognition element for the AP-1 transcription factor complex. We also demonstrated a normal dosage of *kay* was required for lamellocyte formation in *hop^Tum-l^* larvae, since introduction of one mutant allele of the gene encoding the dFos subunit of AP-1 led to a strong suppression of lamellocyte production. One interpretation of these findings is that the lamellocyte enhancer present within *msn* intron-3 serves as a transcriptional target within an activated JNK auto-regulatory circuit that functions to continuously promote the differentiation of lamellocytes from a progenitor hemocyte population (Fig. 8).

A final point of note is that this work has generated numerous *Drosophila* strains that express either *MSNF9-GFP* or *MSNF9-DsRed* selectively in lamellocytes, within the total population of hemocytes present in immune-challenged or genetically-altered larvae. Such transgenes can be used as sensitive probes to assess the role of various genetic loci in the lamellocyte induction process. Additionally, when used in conjunction with the growing collection of *UAS-RNAi* lines designed to selectively inhibit the functions of individual *Drosophila* genes, the *MSNF9-Gal4* strain may prove beneficial as a highly-specific GAL4 driver to newly-discover molecules required for distinct aspects of lamellocyte differentiation and function.

## Materials and Methods

### *Drosophila* strains

*Canton S*, *w^1118^*, *y^1^ w^67c23^*, *y^1^ v^1^ hop^Tum-l^/Basc*, *HeGal4*, *CgGal4*, *lzGal4*; *UAS-EGFP*, *UAS-dTCF^DN^*, *UAS-dRac1*, *UAS-hep^CA^*, *cn^1^ Jra^IA109^ bw^1^ sp^1^/CyO*; *TM6B*, *Sb Tb*, and *CyO*, *ActGFP* were obtained from the Bloomington Stock Center. *kay^1^/TM3*, *Sb^1^ Ser^1^* was provided by D. Bohmann and *UAS-col* was a gift from M. Crozatier. *Bc-GFP* and *eater-GFP* have been previously described [7], [9]. To facilitate the counting of circulating hemocytes in single- or double-heterozygous mutant larvae, *kay^1^* and *Jra^IA109^* were first balanced over the *TM6B, Sb Tb* and *CyO*, *ActGFP* chromosomes, respectively. All flies were maintained at room temperature or 25°C.

### Generation of transgenic *Drosophila* strains for *msn* enhancer analyses

To localize a *msn* DNA region with lamellocyte transcriptional enhancer activity, we used PCR amplification of a *msn*-containing BAC clone to generate nine genomic DNAs that spanned 22 kb of upstream or intronic sequences. These DNAs were sub-cloned into the P-element vectors pH-Stinger [23] and/or pRed H Pelican [24]. Upon the identification of MSNF9-GFP as a DNA transgene active in lamellocytes, but not other blood cell types, we used PCR amplification to generate smaller truncated DNAs for enhancer delimitation. Mutations of potential trans-regulator binding sites were introduced in one of the truncated *msn* DNAs using the QuickChange site-directed mutagenesis kit from Stratagene. After sequence confirmation, altered *msn* DNAs were sub-cloned into the pH Stinger vector. All *msn* enhancer test constructs were injected into *y^1^ w^67c23^* embryos, with multiple transgenic lines generated using standard *Drosophila* transformation procedures [25]. At least five lines were established and analyzed for each DNA tested.

### Hemocyte analyses by fluorescence imaging and cell counting

To monitor and capture GFP and DsRed expression in live animals, third instar larvae were anesthetized with ether, moistened with a small amount of PBS, and viewed using a Zeiss Stereo Lumar fluorescence stereomicroscope. To analyze circulating hemocytes, larvae were bled into 10 µl PBS on a glass slide. Hemolymph samples were allowed to settle for 30 min in a humidity chamber, fixed for 5 min with 4% paraformaldehyde, and incubated in 0.1% TritonX-PBS including Alexa Fluor 594-conjugated phalloidin and DAPI. Images were obtained with a Zeiss Axioplan 2 microscope or an Olympus Fluoview FV500 laser-scanning confocal microscope. To determine the concentration of circulating hemocytes and percent lamellocyte composition therein, we used counting and statistical analyses described previously [26]. For timed development of larvae, egg lays were performed at 25°C for 1–4 hr, and hemolymph samples were obtained and quantified thereafter at times indicated.

### Electrophoretic mobility shift assays

Recombinant proteins containing 6XHis-tagged dJun or dFos DNA-binding domains [27] were purified from *E. coli* by affinity chromatography. Binding reactions were performed in a 20 ml mixture containing 10 mM Tris-HCl (pH 8.0), 10 mM DTT, 2.5 mM MgCl_2_, 10% glycerol, 2 ml poly (dI-dC), 20 mg bovine serum albumin, 100 ng of purified dJun and dFos proteins, and 1,000 cpm of ^32^P-labeled mMTII AP-1 DNA probe [21], with or without wild type or mutant MSNF9 AP-1 oligonucleotide DNAs. DNA fragments were purified and separated on polyacrylamide gels.

### Wasp infestation of *Drosophila* larvae

Batches of six early third-instar larvae were added to 4.5 ml of fly food placed in a 35 mm Petri dish. Three *Leptopilina boulardi* (*L. boulardi* strain *Lb17*) female wasps were added to each batch and allowed to infect larvae for at least 2 hr. A batch of larvae to which no wasps were added was used as a control. After 24 hr, non-infested or infested larvae were removed from the fly food. Wasp encapsulation nodules or lymph glands were dissected from larvae, fixed in 4% paraformaldehyde in PBS, and stained with phalloidin and DAPI. Images of GFP-expressing lamellocytes were obtained with a Zeiss LSM510 confocal microscope.
